# Supplementary material for: Understanding learner behaviour in online courses with Bayesian modelling and time series characterisation
Source: Sci Rep. 2021 Feb 2;11:2823. doi: 10.1038/s41598-021-81709-3 (PMC7854683; doi:10.1038/s41598-021-81709-3)
Supplement: Supplementary file 1 — Supplementary Figures. [file 41598_2021_81709_MOESM1_ESM.pdf]

Supplementary Information: Understanding learner behaviour in online courses with Bayesian modelling and time series characterisation

Robert L. Peach[1,5,*], Sam F. Greenbury [1,2], Iain G. Johnston[3], Sophia N. Yaliraki [4], David Lefevre [5], and Mauricio Barahona [1,*]

[1]Department of Mathematics, Imperial College London, London, UK
[2]NIHR Imperial Biomedical Research Centre, ITMAT Data Science Group, Imperial College London, London, UK
[3]Department of Mathematics, University of Bergen, Bergen, Norway
[4]Department of Chemistry, Imperial College London, London, UK
[5]Imperial College Business School, Imperial College London, London, UK
[*]e-mail correspondence to: r.peach13@imperial.ac.uk; m.barahona@imperial.ac.uk

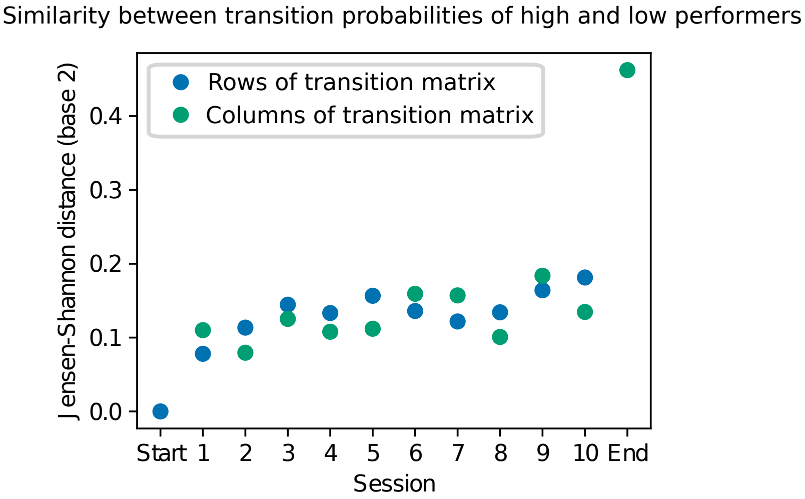

Figure S1. Similarity between the transition probabilities of high and low performers. Here, we look at the similarity (larger Jensen-Shannon distance indicates a larger dissimilarity) between the rows and columns of the transition matrices in Figure **??**. Generally, the similarity is high due to the strong diagonal component of the transition matrices. However, there are clear differences between the high and low performers both with respect to outgoing transitions for a given session (rows) and incoming transitions for a given session (columns). The dissimilarity between the transition patterns of the two groups (high and low performers) increases as the course progresses towards the later sessions.

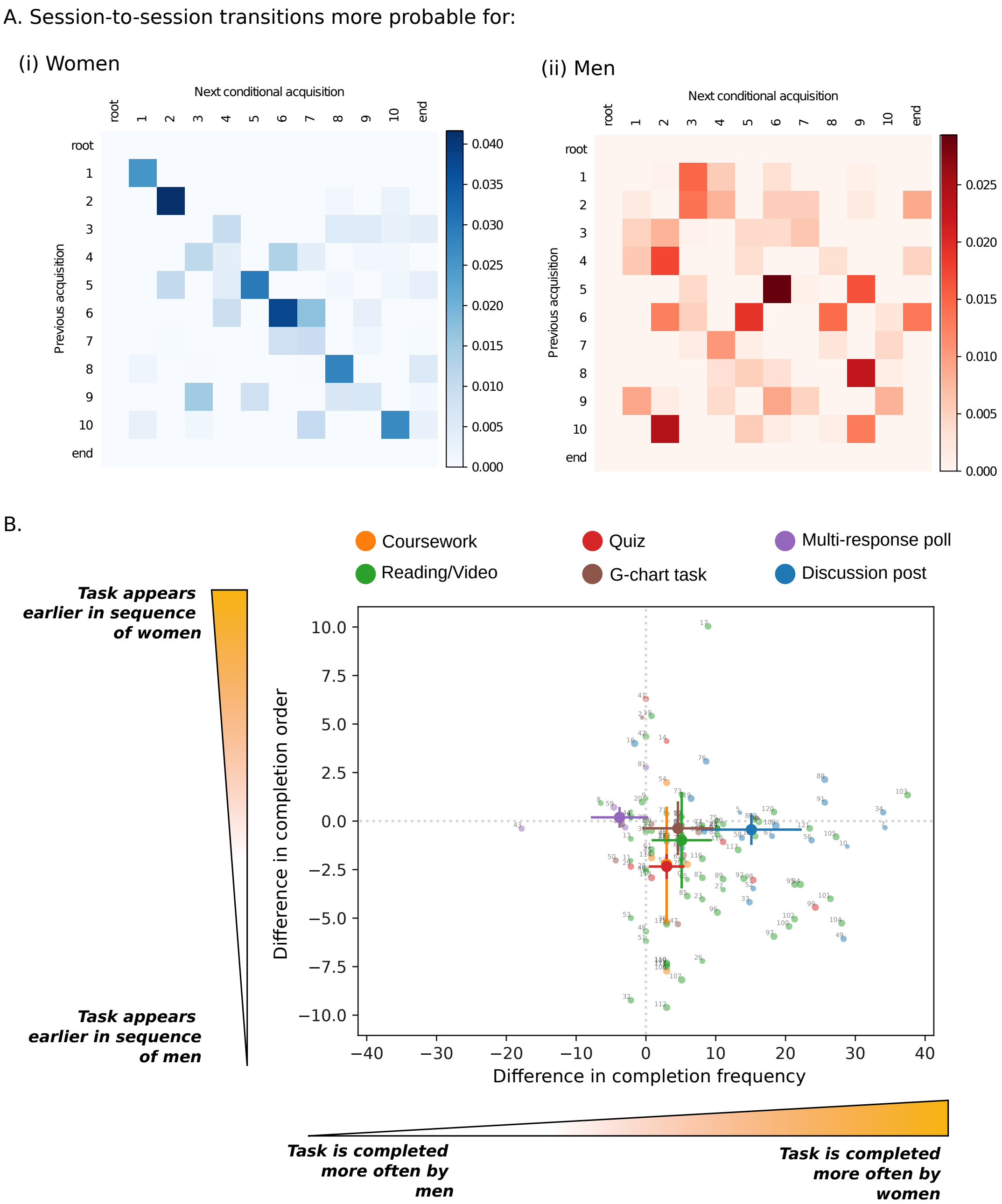

Figure S2. Comparing the male and female groups. (A) The transition probability matrix π between tasks is represented a heatmap (in logarithmic scale) for (i) female and (ii) male learners. Off-diagonal elements correspond to deviations from the course structure. (B) The difference between the two groups in completion frequency versus the difference between the two groups in mean completion order. The tasks are identified by their task ID and colored by type (the six types are listed in the legend). For each task type, the median and interquartile range is plotted.

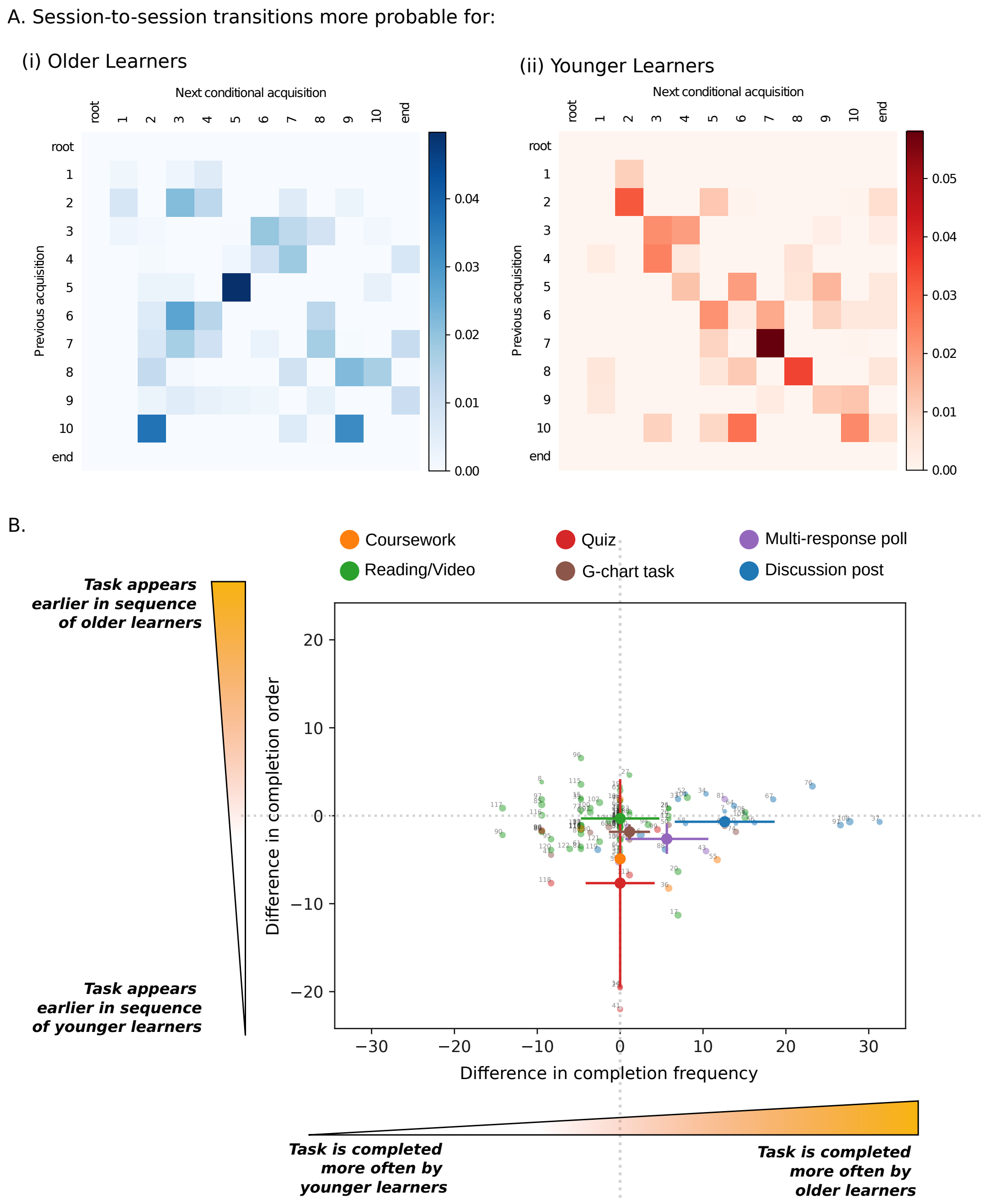

Figure S3. Comparing the ages of learners. (A) The transition probability matrix π between tasks is represented a heatmap (in logarithmic scale) for (i) older (top 25%) and (ii) younger (bottom 25%) learners. Off-diagonal elements correspond to deviations from the course structure. (B) The difference between the older and younger learners in completion frequency versus the difference between the two groups in mean completion order. The tasks are identified by their task ID and colored by type (the six types are listed in the legend). For each task type, the median and interquartile range is plotted.
